# The fate of the rectum in ulcerative colitis at index surgery and beyond—a contemporary cohort

**DOI:** 10.1007/s00384-024-04779-5

**Published:** 2025-01-14

**Authors:** Ian J. B. Stephens, Brenda Murphy, Niamh McCawley, Deborah A. McNamara, John P. Burke

**Affiliations:** 1 Department of Colorectal Surgery, Beaumont Hospital, Dublin 9, Ireland; 2 Royal College of Surgeons Ireland, 123 St. Stephens Green, Dublin, Ireland

**Keywords:** Rectum, Ulcerative colitis, Proctectomy, Ileal pouch-anal anastomosis, Endoscopic surveillance

## Abstract

**Purpose:**

Proctectomy is frequently deferred at index colectomy for ulcerative colitis due to acuity or immunosuppressive treatments. The retained rectum remains symptomatic in over 50% with associated cancer risk. Management options include index or delayed proctectomy with or without restoration of continuity or surveillance. Comparative studies of perioperative outcomes and reasons for retaining the rectum are lacking.

**Methods:**

This 13-year retrospective cohort assesses the fate of the rectum in 168 ulcerative colitis patients by analysing index proctectomy, staged proctectomy and retained rectal remnant determinants and outcomes. The primary outcome was the fate of the rectum. Secondary analysis included perioperative morbidity, length of stay and decision-making determinants.

**Results:**

Proctectomy was performed in 69% of patients, with 16.1% at index surgery. Restorative surgery rate was 44%. Index proctectomy patients were older (54 vs 37 years, *p* < 0.01), more co-morbid (59.3% vs 38.2%, *p* = 0.04) and likely to have elective surgery (81.5% vs 21.3%, *p* < 0.01) or neoplasia (33.3% vs 1.1%, *p* < 0.01). Outcomes after staged proctectomy were comparable, with age influencing restoration of continuity (33.5 vs 46 years, *p* < 0.01). Younger patients were indecisive on proctectomy, while those opting for endoscopic surveillance were older (median 65 years, *p* < 0.01), had more complications (64.3%, *p* = 0.23) and prolonged hospitalisation (median 15 days, *p* = 0.02) at colectomy.

**Conclusions:**

Index proctocolectomy for ulcerative colitis is infrequently performed. Perioperative outcomes of restorative and non-restorative staged proctectomy are comparable. Perioperative experience at colectomy may influence patient decisions regarding future management of their rectum.

**Supplementary information:**

The online version contains supplementary material available at 10.1007/s00384-024-04779-5.

## Introduction

Despite the introduction of biologics in the early 2000s, up to 30% of ulcerative colitis (UC) patients will require surgery either due to refractory chronic or acute severe colitis, dysplasia, malignancy, patient preference or acute complications of colitis such as perforation or toxic megacolon [1–4]. International guidelines advocate for total abdominal colectomy with end ileostomy (TAC) as the operation of choice in the acute setting, or in those patients who cannot safely stop immunosuppressant medication, whereas proctocolectomy with or without restoration of intestinal continuity by ileal pouch-anal anastomosis (IPAA) is recommended in the elective setting where the patient’s pathology and physiology permit and an informed decision regarding restoration of continuity has been made pre-operatively [5–7]. Refractory disease is the most common indication for surgery [8], which is performed emergently in up to 66% of patients [9]. As such, most patients will not have a proctectomy at the time of their colectomy and will have a diverted rectal stump.

The retained rectum will have residual inflammation in 90% of patients, leading to persistent symptoms in over 50% [10, 11]. Patients may undergo further surgery to remove the diseased rectum for symptom management, to treat neoplasia, or to ameliorate rectal cancer risk [10]. The resection may be performed with the formation of an IPAA, or the end ileostomy may be retained (completion proctectomy). Rates of restorative surgery have been extensively investigated internationally [12, 13], as have comparative quality of life outcomes after IPAA and end ileostomy [14]; however, data regarding overall delayed proctectomy rates and comparative patterns of, and outcomes after, restorative and non-restorative surgery largely pre-date modern UC therapies [15–18]. Alternatively, patients may elect to retain their rectal remnant and undergo routine endoscopic surveillance to monitor for dysplasia or malignancy [19]. Though not commonly practiced in the UK and Ireland, ileorectal anastomosis may be considered in motivated patients as a means of avoiding the risks associated with proctectomy balanced against the higher rates of neoplasia [12, 20–22]. When deciding how to proceed after TAC, the patient and surgeon must consider symptom control, quality of life, body image and rectal cancer risk [23]. These are balanced against the risks of pelvic surgery including reduced fecundity and urogenital dysfunction compared to the significant personal and healthcare service commitment required for endoscopic surveillance [24, 25].

This study aims to address the paucity of modern clinical data by comprehensively assessing the fate of the rectum in a typical UC cohort at a tertiary referral centre by characterising determinants of and outcomes following index and delayed proctectomy, staged proctectomy with or without restoration of continuity, and endoscopic surveillance.

## Materials and methods

Patients who underwent elective or emergency total colectomy for inflammatory bowel disease (IBD) with or without proctectomy between January 2010 and June 2023 at a tertiary referral centre (Beaumont Hospital, Dublin) were retrospectively identified by review of physical and electronic theatre logbooks. Cases coded as (sub)total colectomy with end ileostomy or ileorectal anastomosis, (pan)proctocolectomy with end ileostomy or proctocolectomy with IPAA were eligible for inclusion. Patients aged 18 years and older undergoing surgery for UC were taken forward for comparative analysis. Patients with Crohn’s disease, indeterminant colitis, prior segmental colectomy or proctectomy were excluded. Patient demographics, perioperative details, subsequent surgeries and clinical follow-up were established using hospital information systems, chart review and clinical or phone call follow-up as required. This review was registered with the institutional clinical audit office (audit registration number CA2023/224).

The primary outcome of interest was the fate of the rectum. This was categorised as (1) index proctectomy defined as either proctocolectomy with end ileostomy or IPAA at the time of primary surgery, (2) staged proctectomy defined as either subtotal colectomy with end ileostomy followed by completion proctectomy alone or completion proctectomy with IPAA or (3) rectum in situ. For patients whose rectum remained in situ, it was determined whether patients were awaiting surgery, elected to pursue endoscopic surveillance alone or were undecided regarding future surgery. If patients had not been followed within the last year, they were contacted by telephone or offered a clinic appointment to establish a management stream. For patients who had transferred their care to another institution, further surgery was determined by phone call or contact with their general practitioner or treating institution.

Demographic data including age, gender, diagnosis, co-morbidities, steroid and biologic use was extracted from medical charts and electronic patient records. Perioperative data included indication for surgery, timing of surgery, rates of minimally invasive surgery, conversion to open and post-operative length of stay (LoS). Clavien Dindo Classification [26] and Comprehensive Complication Index (CCI) [27] were used to classify 30-day morbidity and mortality outcomes. Obstruction, perforation and toxic megacolon were categorised as acute complications under indication. Timing of surgery was defined as “elective”, “urgent” or “immediate” based on whether it was planned pre-hospital, performed on a hospitalised patient after failure of medical management or due to the development of an acute complication respectively [9, 28].

Index and delayed proctectomy were compared based on patient demographics and perioperative variables from the time of index surgery. An analysis of the delayed proctectomy group was performed by comparing patient demographics, perioperative colectomy outcomes and post-proctectomy outcomes between restorative (3-stage IPAA) and non-restorative (completion proctectomy) surgeries performed at Beaumont Hospital. Finally, these parameters were assessed in the rectum in situ group, with the inclusion of endoscopic follow-up data.

Statistical analysis was performed using STATA® [29]. Descriptive statistics are reported as median (interquartile range) unless stated otherwise. Shapiro–Wilk test was used to assess the distribution of continuous outcomes. Univariable analysis was performed using the Mann–Whitney *U* test for non-parametric data and Fisher’s exact test for parametric data. The Kruskal–Wallis test was used for continuous variables.

## Results

**Fig. 1:**
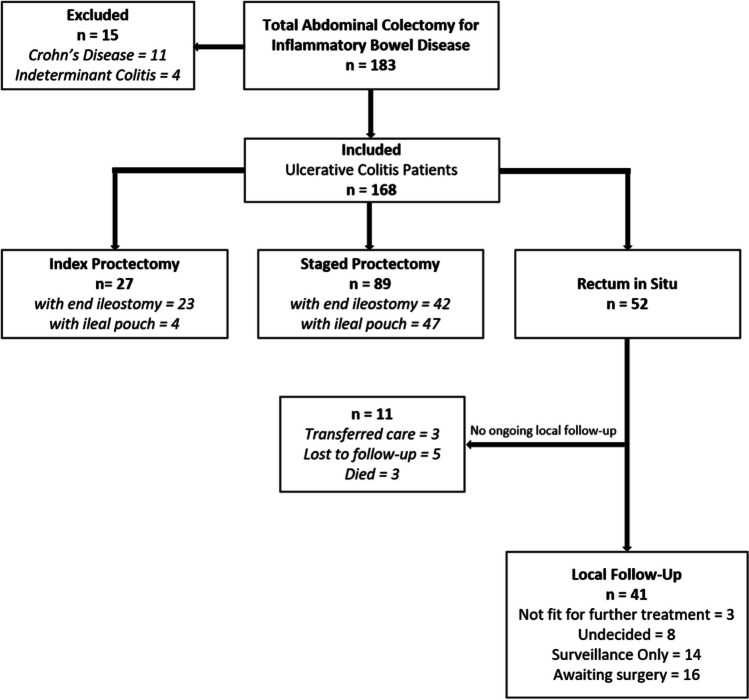
A total of 183 patients underwent total abdominal colectomy between January 2010 and June 2023 for inflammatory bowel disease. Following the application of inclusion criteria, 168 ulcerative colitis patients were identified. *n* = total patient number per group, with subgroup numbers denoted in italics. Below these outcomes, follow-up attrition and surgical preferences of patients with their rectum still in situ are reported

Over the 13-year period investigated, 168 total colectomies were performed for UC. Index proctectomy was performed in 27 (16.1%) patients, and 89 (53%) proceeded to a staged proctectomy. Of the 116 rectal resections performed, 51 (44%) included restoration of intestinal continuity by either 2-stage (4) or 3-stage (47) IPAA. A total of 14 patients had their proctectomy performed at another hospital (7 IPAA, 7 completion proctectomy). The rectum remains in situ for 52 patients, 11 of whom are no longer being followed in this institution (Fig. 1).

### Timing of proctectomy

Patients that underwent index proctectomy were significantly older (median age 54 vs 37 years, *p* < 0.01), more likely to have co-morbidity (59.3% vs 38.2%, *p* = 0.04) and were significantly less likely to be taking steroids (37% vs 87.6%, *p* < 0.01) or biologics (37% vs 75.3%, *p* = < 0.01) in the 12 weeks preceding surgery. Cardiovascular disease and pre-existing malignancy were more common in the proctocolectomy group (29.6% vs 7.9%, *p* = 0.01, 18.5% vs 1.1%, *p* < 0.05) (Supplemental Table 1). Index proctectomy was more commonly performed as elective surgery (81.5% vs 21.3%, *p* < 0.01), for neoplasia (33.3%% vs 1.1%, *p* < 0.01) or chronic refractory colitis (48.2% vs 18% *p* < 0.01) than staged proctectomy. Conversely, it was less likely performed for acute refractory colitis (18.5% vs 70.8%, *p* < 0.01) or as urgent surgery (18.5% vs 70.8%, *p* < 0.01). Restorative surgery was much more prevalent in delayed proctectomy cases (52.8% vs 14.8%, *p* < 0.01) (Table 1). Post-operative morbidity was 34.5% across the entire cohort, but higher in those that had an index proctectomy (40.7%) compared to those that had staged surgery (32.6%). Surgical site infection (SSI) was the most common complication, at 14.7%, followed by venous thromboembolic events (VTE)(4.3%), and bleeding requiring transfusion or reoperation (5.1%). Ileus and high stoma output were more common in the index proctectomy group (3.7% vs 3.4% and 7.4% vs 1.1%, respectively) (Supplemental Table 2).

**Table 1:** Patient demographics and perioperative clinical parameters at the time of total abdominal colectomy for index and staged proctectomy groups

	Overall	Index proctectomy	Staged proctectomy	*P *value
	(*n=*116)	(*n* = 27)	(*n* = 89)	
Patients				
Female	46 (39.7%)	9 (33.3%)	37 (41.6%)	0.51
Male	70 (60.3%)	18 (66.7%)	52 (58.4%)	
Age at colectomy (years)	39.5 (31.5–53)	54 (45–64)	37 (30–48)	< ***0.01***
Timing of colectomy				
Elective	41 (35.4%)	22 (81.5%)	19 (21.3%)	< ***0.01***
Urgent	68 (58.6%)	5 (18.5%)	63 (70.8%)	< ***0.01***
Immediate	7 (6%)	0 (0%)	7 (7.9%)	0.30
Indication for colectomy				
Acute refractory colitis	68 (58.6%)	5 (18.5%)	63 (70.8%)	< ***0.01***
Chronic refractory colitis	29 (25%)	13 (48.2%)	16 (18%)	< ***0.01***
Neoplasia	10 (8.6%)	9(33.3%)	1 (1.1%)	< ***0.01***
Acute complication	9 (7.8%)	0(%)	9 (10.1%)	0.20
Treatment within 12 weeks of colectomy				
Steroids	88 (75.9%)	10 (37%)	78 (87.6%)	< ***0.01***
Biologics	77 (66.4%)	10 (37%)	67 (75.3%)	< ***0.01***
Outcomes at colectomy				
Minimally invasive surgery	95 (81.9%)	18 (66.7%)	77 (86.5%)	***0.04***
Length of stay	7 (5.5–10)	9 (7–13)	7 (5–9)	< ***0.01***
30-day morbidity	40 (34.5%)	11 (40.7%)	29 (32.6%)	0.29
Restorative surgery	51 (44.0%)	4 (14.8%)	47 (52.8%)	< ***0.01***

Age at colectomy reports median age in years (interquartile range); length of stay reports median in days (interquartile range). All other values listed are *n* (%) where *n* = patient number, and % = percentage of group. Bold Italic text signifies a statistically significant *p* value

### Staged proctectomy outcomes

Post-operative outcome data was available for the 75 staged proctectomies performed at this institution. Patients undergoing delayed restorative surgery by proctectomy with IPAA formation were younger than those who had a completion proctectomy with end ileostomy (median age 33.5 (26–39) vs 46 (39–56) years, *p* < 0.01) and were less likely to have co-morbidity (27.5% vs 48.6%, *p* = 0.05). This was primarily attributable to rates of cardiovascular disease between the groups (17.1% vs 2.5%, *p* = 0.07). The only patient with primary sclerosing cholangitis had a 3-stage IPAA, and the 7 patients with extraintestinal manifestations of IBD were split between the completion proctectomy and pouch groups (Supplemental Table 1). Neither perioperative variables that were related to deferral of proctectomy at the time of TAC nor post-operative outcomes following index surgery showed an association with restorative surgery. The definitive reason for a patient undergoing a completion proctectomy rather than a pouch was not documented in 12 of 35 (34.3%) cases. In the remainder, it was due to patient preference (39.1%) or physician recommendation based on co-morbidity (21.9%), obesity (8.7%), anal sphincter function (8.7%), operative technical factors (8.7%), chronic anal pathology (4.3%), patient functional status (4.3%) or surgical history (4.3%).

Outcomes after both staged proctectomy with or without IPAA were comparable with median LoS of 6 (5–9) and 7 (6–10) days, 30-day post-operative morbidity rates of 40% and 42.9% and rates of minimally invasive surgery of 72.5% and 77.1% respectively (Table 2). Superficial SSI was the most common complication at 17.3%, followed by deep SSI (6.7%), bleeding events (4%) and anastomotic complications (4%) (Supplemental Table 2). The histological assessment showed evidence of active inflammation in almost all samples with features of predominately chronic UC (37.3%), diversion proctocolitis (25.3%) or mixed diversion and inflammatory proctocolitis (28%). There was a single post-operative diagnosis of Crohn’s disease in the completion proctectomy cohort and quiescent UC in only 3 patients. The patient that had a delayed proctectomy for dysplasia had no evidence of dysplastic changes in their final specimen, only features of chronic UC.

**Table 2:** Demographics, perioperative colectomy variables, and post-operative outcomes for staged proctectomy with subgroup analysis comparing completion proctectomy and 3-stage IPAA^1^

	Overall	Completion proctectomy	3—Stage IPAA^1^	*P* value
	(***n*** = 75)	(***n*** = 35)	(***n*** = 40)	
Patient demographics				
Female	31 (41.3%)	17 (48.6%)	14 (35%)	0.25
Male	44 (58.7%)	18 (51.4%)	26 (65%)	
Age at proctectomy (years)	39 (33–49)	46 (39–56)	33.5 (26–39)	***< 0.01***
Total colectomy perioperative variables				
Age at colectomy (years)	36 (30–46)	44 (36–56)	31 (24–37)	***<0.01***
Timing of colectomy				
Urgent	52 (69.3%)	26 (74.3%)	26 (65%)	0.46
Immediate	5 (6.7%)	0 (0%)	5 (12.5%)	0.03
Elective	18 (24%)	9 (25.7%)	9 (22.5%)	0.95
Indication for colectomy				
Acute refractory colitis	52 (69.4%)	27 (77.1%)	25 (62.5%)	0.21
Chronic refractory colitis	15 (20%)	7 (20%)	8 (20%)	0.98
Dysplasia	1 (1.3%)	0 (0%)	1 (2.5%)	0.35
Acute complication	7 (9.3%%)	1 (2.9%)	6 (15%)	***0.04***
Treatment within 12 weeks of colectomy				
Steroids	64 (85.3%)	29 (82.9%)	35 (87.5%)	0.81
Biologics	56 (74.7%)	25 (71.4%)	31 (77.5%)	0.74
30-day post-colectomy morbidity	26 (34.7%)	15 (42.9%)	11 (27.5%)	0.25
CCI^2^	25.5 (± 14.8)	25.4 (± 15.8)	25.6 (± 14.2)	0.73
Minimally invasive colectomy	64 (85.3%)	28 (80%)	36 (90%)	0.85
Length of stay after colectomy (days)	7 (5–10)	7 (6–10)	6 (5–9)	0.31
Outcomes at proctectomy				
30-day post-proctectomy morbidity	31 (41.3%)	15 (42.9%)	16 (40%)	0.49
Clavien Dindo I–II	24 (32%)	12 (34.3%)	12 (30%)	0.81
Clavien Dindo III +	7 (9.3%)	3 (8.6%)	4 (10%)	0.83
CCI^2^	21.9 (± 8.8)	21.9 (± 9.4)	21.8 (± 8.5)	0.75
Reoperation	6 (8%)	2 (5.7%)	4 (10%)	0.81
Minimally invasive proctectomy	56 (74.7%)	27 (77.1%)	29 (72.5%)	0.85
Conversion to open	4 (5.3%)	1 (2.9%)	3 (7.5%)	0.72
Length of stay after proctectomy (days)	7 (5 to 9)	7 (5–10)	6 (4.5–8)	0.77

^1^Ileal Pouch-Anal Anastomosis Measures of time are reported as median (interquartile range) ^2^Comprehensive Complication Index, reported as mean (± standard deviation) All other values listed are *n* (%) where *n* = patient number, and % = percentage of group. Bold Italic text signifies a statistically significant *p* value

### Rectum in situ

Of the 52 patients that have not had a proctectomy to date, 3 have died, 3 have transferred to another institution and 5 have been lost to follow-up. A total of 41 are being actively followed, with 3 considered unfit for further intervention. The remaining 38 fall into three categories—awaiting surgery, endoscopic surveillance only or undecided. Age, both current and at colectomy, and LoS after colectomy were the only features that differ significantly between these groups. The patients opting for surveillance only are older (median age 65 vs 38 vs 28.5 years, *p* < 0.01) and remained in the hospital longer after colectomy (median LoS 15 vs 8.5 vs 8 days, *p* = 0.02) than those awaiting surgery or undecided respectively. There is also a higher portion of patients who experienced post-colectomy morbidity in the surveillance group (64.3% vs 31.3% vs 37.5%, *p* = 0.23). Of note, 7 patients are awaiting pouch surgery, and 9 non-restorative proctectomy. There are 4 patients deferring these operations until they have completed their families, and 3 more due to social circumstances or clinical optimization. Endoscopy is up to date in 68.4% of patients, with a median of 16 (7–38) months since the last rectal endoscopy (Table 3).

**Table 3:** Clinical demographics, perioperative colectomy, and surveillance data for patients with rectum in situ under active management

	Overall	Awaiting surgery	Surveillance only	Undecided	*P* values
	(*n*=38)	(*n*=16)	(*n*=14)	(*n*=8)	
Demographics					
Current age (years)	44 (32–60)	38 (32.5–48)	65 (56–70)	28.5 (21.5–36.5)	< ***0.01***
Female	16 (42.1%)	6 (37.5%)	5 (35.7%)	5 (62.5%)	0.45
Male	22 (57.9%)	10 (62.5%)	9 (64.3%)	3 (37.5%)	
Time since colectomy (months)	45 (7–38)	46.5 (33–88)	47 (36–79)	37.5 (21.5–49)	0.17
Age at colectomy (years)	38 (27–56)	33 (27–41)	61 (45–66)	24.5 (19.5–34)	< ***0.01***
Timing of colectomy					
Elective	5 (13.2%)	2 (12.5%)	1 (7.1%)	2 (25%)	0.60
Immediate	2 (5.3%)	1 (6.3%)	1 (7.1%)	0 (0%)	0.75
Urgent	31 (81.5%)	13 (81.2%)	12 (85.8%)	6 (75%)	0.87
Indication for colectomy					
Acute refractory colitis	32 (84.2%)	13 (81.2%)	12 (85.8%)	7 (87.5%)	0.91
Chronic refractory colitis	3 (7.9%)	2 (12.5%)	1 (7.1%)	0 (0%)	0.79
Neoplasia	1 (2.6%)	0 (0%)	0(0%)	1 (12.5%)	0.21
Acute complication	2 (5.3%)	1 (6.3%)	1 (7.1%)	0 (0%)	0.75
30-day post-operative morbidity	17 (44.7%)	5 (31.3%)	9 (64.3%)	3 (37.5%)	0.23
Clavien Dindo I-II	15 (39.5%)	4 (25%)	8 (57.1%)	3 (37.5%)	0.19
Clavien Dindo III +	2 (5.3%)	1 (6.3%)	1 (7.1%)	0 (0%)	0.75
Minimally invasive colectomy	36 (94.7%)	15 (93.8%)	13 (92.9%)	8 (100%)	0.86
Length of stay after colectomy (days)	9.5 (7–16)	8.5 (6.5–13)	15 (9–19)	8 (5.5–10)	***0.02***
Endoscopic follow-up					
Time since last rectal endoscopy	16 (7 −38)	14 (7–28)	21.5 (6 to 52)	18 (6.5–31.5)	0.67
Rectal endoscopy within < 2 yrs	26 (68.4%)	12 (75%)	8 (57.1%)	6 (75%)	0.60

Measures of time are reported as median (interquartile range) Values listed are *n* (%) where *n* = patient number and % = percentage of group. Bold Italic text signifies a statistically significant *p* value

## Discussion

This study gives a comprehensive assessment of the fate of the rectum in a typical UC colectomy cohort. A total of 116 patients, from the original 168 UC total colectomy group, had a proctectomy performed either as part of primary resection or as a staged procedure. In these patients, there was a tendency towards non-restorative surgery with 56% of patients undergoing an index proctocolectomy with end ileostomy or total colectomy with end ileostomy followed by completion proctectomy. The remaining 44% underwent restorative proctocolectomy with IPAA either as a two-stage or three-stage procedure. For the patients who still have their rectal remnant in situ, 41 are being followed at this institution. The majority of these have opted for further surgery with a similar pattern of preference regarding restoration of intestinal continuity (43.8% restorative). Endoscopic surveillance was up-to-date in over two-thirds of those fit for it.

Where possible, index proctectomy with or without IPAA is preferable as it allows for the removal of the entirety of the diseased colon and rectum at a single operation; however, this cohort demonstrates that due to high rates of refractory disease, dependence on medical therapy and frequent need for emergent surgery, it is infrequently performed (16.1%). TAC is the procedure of choice in the acute colitic and those who cannot stop immunosuppressive medical therapy, or in patients who have not definitively decided if they wish to have a pouch, but this approach leaves the diseased rectum in situ unless patients undergo further surgery which in turn carries added morbidity [5–7]. As a result, significant numbers of patients will continue to have troublesome symptoms [18, 30], most typically bloody discharge [10] with up to 55% requiring topical treatment [11]. Other complications of chronic proctitis include stricturing, fistulation, abscess formation, profuse bleeding, systemic symptoms of IBD and neoplasia [18]. The lifetime risk of neoplasia in the diverted rectal stump is estimated at 2.2% at 20 years or 6.5 cases per 100,000 patient-years based on both primary cohort studies and meta-analysis [21, 31]. Though reduction of cancer risk is often discussed when counselling patients on the risk and benefits of proctectomy, it is symptom control that drives most patients to opt for further surgery [11, 30]. The results reported here support this, with high rates of active or chronic inflammation demonstrated at histological review, and no evidence of neoplasia in the staged surgery subgroup.

European guidelines advocate for IPAA as the procedure of choice after TAC, though recognise that permanent end ileostomy may be be reasonable for some patients [6]. Systematic review has demonstrated that quality of life (QoL) after IPAA and permanent end ileostomy are comparable, with reconstructive surgery scoring better on body image indexes [14]. Rates of restorative surgery vary significantly both internationally and between hospitals with an international comparison of restorative surgery following TAC in UC between England and Sweden showing significant differences in rates of restoration of intestinal continuity, 33% and 46%, respectively. Furthermore, there were stark differences in the operation of choice, with 92.3% of English patients having pouch surgery, compared to 38.8% in Sweden with the remainder undergoing ileorectal anastomosis [12]. Similarly, significant differences exist between hospitals within a region. In New York, IPAA rates after colectomy vary from 7 to 63% across hospitals [13]. The overall rate of restorative surgery at the time of proctectomy in this institute was 44% but reached 53.3% in the patients undergoing delayed proctectomy.

Overall proctectomy rates after TAC vary significantly from 27 to 92.6% [11, 15, 16, 30, 32], though several of these studies pre-date IPAA and biologics. Modern cohorts, such as Munie 2013, Porter 2021 and Boldovjakova 2023, give a breakdown of rectal fate, with overall proctectomy (76.9%, 27%, 65.7%) and IPAA rates (65.7%, 16.7%, 17.2%) [11, 30, 32]. Proctocolectomy as either a single or staged procedure carries a significant risk of morbidity with recent rates reported ranging from 16.4 to 50.4% [11, 30, 33]. Outcomes at the time of staged proctectomy reported here demonstrate high rates of minimally invasive surgery, comparable rates of post-operative morbidity and short LoS with no significant differences between patients undergoing restorative and non-restorative surgery. Of the post-operative morbidity reported here (41.3%), over half was attributable to SSIs (24%), and less than 25% constituted major (Clavien Dindo III +) complications. Of the SSIs, 72% were superficial.

Patients who underwent IPAA were significantly younger than those who continued with an end ileostomy. Despite good evidence suggesting acceptable perioperative and functional QoL outcomes after IPAA in well-selected, older patients [34, 35], our experience locally has been that few elderly chose this operation when offered. Of the patients who underwent proctectomy without reconstruction, almost 40% opted to do so based on personal preference despite being clinically suitable for pouch surgery. No patients were disqualified from pouch surgery based on complications at TAC, and the only patient with a rectal stump complication underwent a 3-stage IPAA without event. Similar to our findings, a recent US analysis demonstrated a 50.2% IPAA rate in insured patients with UC undergoing either index or delayed proctectomy, with younger age, elective surgery and laparoscopic approach being significantly associated with IPAA formation. However, the lower rates of permanent end ileostomy (16.8% vs 38.6%) and higher rates of patients not undergoing further surgery (33% vs 26.8%) compared to ours likely reflect the exclusion of over 65 s and patients without continuous insurance [36]. When limiting our analysis to under 65 s, the overall rate of IPAA is 46.8%, with 36.7% having a proctocolectomy with end ileostomy and 16.5% having their rectum in situ, respectively.

Age and length of post-operative hospitalisation were associated with management preference for patients who still retain their rectal remnant. Seventy-five per cent of the undecided cohort were under the age of 36.5 years. This age group may have particularly strong concerns regarding fecundity, body image and sexual function and hence have reservations around further surgery. In contrast, patients electing to continue with only endoscopic surveillance were significantly older and had a period of prolonged hospitalisation at colectomy and commonly experienced a post-operative complication. As such, they may be more willing to accept symptoms and risk of neoplasia against their personal prior experience of major abdominal surgery.

Specific guidelines regarding surveillance of the diverted rectum in IBD are varied, recommending typically 6–24 monthly intervals [37] and compliance with surveillance is poor, and can be as low as 40% [10, 19, 32, 38]. International guidelines have advocated for a risk-based approach to surveillance, with interval based on duration of disease, previous history of neoplasia, degree of inflammation, extent of disease, presence of inflammatory polyps, strictures, dysplasia, background of primary sclerosing cholangitis and family history of colorectal cancer [5, 7]. Of the patients under active management in our cohort, 68.4% had been endoscoped within the last 2 years.

The limitations of this study are its retrospective, non-randomised nature. Individual surgeon and/or institutional practices may have influenced the choice of operation both at index and subsequent surgery. As discussed above, significant variation in IPAA rates exists between hospitals, with surgeon-level factors explaining up to 21% of this variation [13]. Our practice would be such that in the absence of significant co-morbidity or contraindication to IPAA all patients are counselled on the risks and benefits of both restorative and non-restorative surgery as part of a shared decision-making process.

This retrospective TAC cohort outlines the management of the rectum in UC surgery and adds significant data to support multidisciplinary team and patient decision-making. The inclusion of all fates for the rectum (index proctectomy, staged proctectomy, rectum in situ) with comparative clinical outcome data and analysis of the clinical determinants of decision-making allows this article to give a holistic, pragmatic overview of management choices for the rectum in UC surgery.

## Conclusion

Index proctocolectomy has a limited but safe role in ulcerative colitis surgery. Outcomes at the time of colectomy may influence the choice of restorative or non-restorative staged proctectomy, but younger patients tended towards IPAA. Post-operative outcomes for both these procedures were comparable, and high rates of restorative surgery are achievable safely. For patients whose rectum remains in situ, patterns were demonstrated among groups. Younger patients are less inclined to make decisions regarding further surgery, and those opting for surveillance alone are older and experienced worse perioperative colectomy outcomes. Significant international variation in proctectomy rates, restorative surgery and endoscopic follow-up after TAC highlight the need for multinational UC registries to aid in the study and management of these complex patients.

## Supplementary information

Below is the link to the electronic supplementary material.

Supplementary Material (DOCX 23.5 KB)

## Data Availability

Data will be provided by authors upon reasonable request.
