# Supplementary material for: The fate of the rectum in ulcerative colitis at index surgery and beyond—a contemporary cohort
Source: Int J Colorectal Dis. 2025 Jan 14;40(1):12. doi: 10.1007/s00384-024-04779-5 (PMC11732862; doi:10.1007/s00384-024-04779-5)
Supplement: Supplementary file 1 — Supplementary Material (DOCX 23.5 KB) [file 384_2024_4779_MOESM1_ESM.docx]

| **Supplemental Table 1 –** Patient co-morbidity by (S1.1) approach to staging of surgery and (S1.2) reconstruction at delayed proctectomy | | | |  |
| --- | --- | --- | --- | --- |
| **S1.1 - Co-morbidities for Index compared to Delayed Proctectomy** | | | | |
|  | **Overall Cohort (n = 116)** | **Index Proctectomy (n = 27)** | **Delayed Proctectomy (n = 89)** |  |
| **Co-morbidity Category** |  |  |  | **P value** |
| Any | 50(43.1) | 16(59.3) | 34(38.2) | ***0.04*** |
| Previous abdominal surgery | 7(6) | 2(7.4) | 5(5.6) | 0.99 |
| Cardiovascular disease | 15(12.9) | 8(29.6) | 7(7.9) | ***0.01*** |
| Diabetes mellitus | 4(3.4) | 2(7.4) | 2(2.2) | 0.46 |
| Respiratory | 6(5.2) | 2(7.4) | 4(4.5) | 0.85 |
| Autoimmune disease | 13(11.2) | 1(3.7) | 12(13.5) | 0.28 |
| Endocrine disorder | 10(8.6) | 2(7.4) | 8(9) | 0.99 |
| Malignancy | 6(5.2) | 5(18.5) | 1(1.1) | ***<0.05*** |
| Genitourinary pathology | 5(4.3) | 2(7.4) | 3(3.4) | 0.66 |
| Mood disorder | 6(5.2) | 0(0) | 6(6.7) | 0.39 |
| Primary sclerosing cholangitis | 2(1.7) | 1(3.7) | 1(1.1) | 0.83 |
| Extraintestinal manifestation of ulcerative colitis | 8(6.9) | 0(0) | 8(9) | 0.22 |
|  |  |  |  |  |
| **S1.2 Co-morbidities for Completion Proctectomy compared to IPAA^1^ in Delayed Cohort** | | | | |
|  | **Delayed Proctectomy (n = 75)** | **Completion Proctectomy (n = 35)** | **3-Stage IPAA^1^ (n = 44)** |  |
| **Co-morbidity Category** |  |  |  | **P value** |
| Any | 28(37.3) | 17(48.6) | 11(27.5) | ***0.05*** |
| Previous abdominal surgery | 2(2.7) | 2(5.7) | 0(0) | 0.44 |
| Cardiovascular disease | 7(9.3) | 6(17.1) | 1(2.5) | 0.07 |
| Diabetes mellitus | 2(2.7) | 2(5.7) | 0(0) | 0.43 |
| Respiratory | 3(4) | 2(5.7) | 1(2.5) | 0.89 |
| Autoimmune disease | 11(14.7) | 6(17.1) | 5(12.5) | 0.81 |
| Endocrine disorder | 7(9.3) | 4(11.4) | 3(7.5) | 0.85 |
| Malignancy | 1(1.3) | 0(0) | 1(2.5) |  |
| Genitourinary pathology | 2(2.7) | 2(5.7) | 0(0) | 0.43 |
| Mood disorder | 6(8) | 2(5.7) | 4(10) | 0.81 |
| Primary sclerosing cholangitis | 1(1.3) | 0(0) | 1(2.5) |  |
| Extraintestinal manifestation of ulcerative colitis | 7(9.3) | 4(11.4) | 3(7.5) | 0.85 |
| 1 = Ileal Pouch-Anal Anastomosis. Values listed are n (%) were n = patient number, and % = percentage of group. | | | | |

| **Supplemental Table 2 –** Complications occurring within 30-days of (S2.1) total abdominal colectomy with or without index proctectomy and (S2.2) at staged proctectomy with or without IPAA^1^ | | | |
| --- | --- | --- | --- |
| **S2.1 - Complications at Total Abdominal Colectomy** | |  |  |
|  | **Total Abdominal Colectomy (n = 116)** | **With Proctectomy (n = 27)** | **Without Proctectomy (n = 89)** |
| **Patient No.** | 40(34.5%) | 11(40.7%) | 29(32.6%) |
| **Complication Category** |  |  |  |
| Surgical site infection | 17(14.7%) | 4(14.8%) | 13(14.6%) |
| *Superficial* | *7(6%)* | *2(7.4%)* | *5(5.6%)* |
| *Deep* | *10(8.6%)* | *2(7.4%)* | *8(9%)* |
| Venous thromboembolism | 5(4.3%) | 2(7.4%) | 3(3.4%) |
| Bleeding | 6(5.1%) | 2(6.4%) | 4(5.5%) |
| Ileus | 4(3.4%) | 1(3.7%) | 3(3.4%) |
| Urinary Retention | 3(2.6%) | 1(3.7%) | 2(2.2%) |
| High output Stoma | 3(2.6%) | 2(7.4%) | 1(1.1%) |
| COVID-19 | 2(1.7%) | 0(0%) | 2(2.2%) |
| Pneumonia | 2(1.7%) | 0(0%) | 2(2.2%) |
| Small bowel obstruction | 2(1.7%) | 1(3.7%) | 1(1.1%) |
| TPN | 1(0.9%) | 0(0%) | 1(1.1%) |
| Arrhythmia | 1(0.9%) | 0(0%) | 1(1.1%) |
| Intraoperative | 1(0.9%) | 0(0%) | 1(1.1%) |
| **Total complications** | 47 | 13 | 34 |
| **S2.2 Complications at Staged Proctectomy** | |  |  |
|  | **Delayed Proctectomy (n = 75)** | **Completion Proctectomy (n = 35)** | **3-Stage IPAA^1^ (n = 44)** |
| **Patient No.** | 31(41.3%) | 15(42.9%) | 16(36.4%) |
| **Complication Category** |  |  |  |
| Surgical site infection | 18(24%) | 7(20%) | 11(25%) |
| *Superficial* | *13(17.3%)* | *6(17.1%)* | *7(15.9%)* |
| *Deep* | *5(6.7%)* | *1(2.9%)* | *4(9.1%)* |
| Small bowel obstruction | 2(2.7%) | 1(2.9%) | 1(2.3%) |
| Urinary tract infection | 2(2.7%) | 2(5.7%) | 0(0%) |
| Urinary retention | 2(2.7%) | 2(5.7%) | 0(0%) |
| Bleeding | 3 (4%) | 3(8.6%) | 0(0%) |
| Pneumonia | 2(2.7%) | 1(2.9%) | 1(0%) |
| Arrhythmia | 1(1.3%) | 1(2.9%) | 0(0%) |
| Ileus | 1(1.3%) | 0(0%) | 1(2.3%) |
| Anastomotic Complication | 3(4%) | 0(0%) | 3(6.8%) |
| Fascial dehiscence | 1(1.3%) | 1(2.9%) | 0(0%) |
| Post epidural headache | 1(1.3%) | 1(2.9%) | 0(0%) |
| **Total complications** | 36 | 19 | 17 |
| 1 = Ileal Pouch-Anal Anastomosis. Values listed are n (%) were n = patient number, and % = percentage of group. | | | |
